# Role of AMP-Activated Protein Kinase in the Control of Appetite

**DOI:** 10.1111/j.1365-2826.2008.01745.x

**Published:** 2008-07

**Authors:** B Kola

**Affiliations:** Centre for Endocrinology, Barts and the London School of Medicine and Dentistry, University of LondonLondon, UK

**Keywords:** AMPK, feeding, hypothalamus, appetite, hypoglycaemia, insulin, ghrelin, leptin, adiponectin

## Abstract

AMP-activated protein kinase is a key enzyme in the regulation of energy metabolism. Its activation has pleiotropic effects in multiple tissues, including increased fatty acid oxidation, glucose uptake and glycolysis, as well as the inhibition of fatty acid and glycogen synthesis and gluconeogenesis, and stimulation of mitochondrial biogenesis. Recently, the AMP-activated protein kinase (AMPK) has also emerged as a regulator of appetite, contributing to the control of energy metabolism at both cell and the whole body levels. Pharmacological and genetic activation or inhibition of hypothalamic AMPK lead to increased or reduced food intake, respectively. AMPK appears to play a role in hypothalamic glucose and nutrient sensing and numerous studies have suggested a role for AMPK in mediating the orexigenic or anorexigenic effects of various endogenous and exogenous substances.

The AMP-activated protein kinase (AMPK) is an evolutionarily conserved enzyme that senses the energy status of the cell and regulates fuel availability. AMPK is a heterotrimeric protein consisting of catalytic α and regulatory β and γ subunits; it is activated allosterically by an increase in the intracellular AMP/ATP ratio as well as by phosphorylation on Thr172 by upstream kinases (1). Two upstream AMPK kinases have been previously identified: LKB1 and Ca^2+^/calmodulin-dependent protein kinase kinase (CaMKK) (2, 3). CaMKKβ is expressed primarily in the brain and therefore the Ca^2+^mediated pathway may be the most relevant in neurones (4). Recently, transforming growth factor-β-activated kinase (TAK1), a member of the mitogen-activated protein kinase kinase kinase family, has also been proposed as a candidate AMPK kinase in mammalian cells (5). AMPK is ubiquitously expressed and plays a major role in fatty acid and glucose metabolism (6). AMPK activity in liver, fat, skeletal muscle and other tissues is affected by numerous compounds (7) and activation of this enzyme, via stimulation of catabolic pathways and inhibition of anabolic pathways, results in an improved lipid and glucose profile and in an improved insulin-sensitivity status of the whole organism (1).

The role of AMPK in the regulation of body weight and energy homeostasis is not limited to its actions in the peripheral tissues. In recent years, AMPK has emerged as a nutrient and glucose sensor in the hypothalamus (8, 9) and its role in appetite regulation has been thoroughly investigated by several groups. AMPK is widely expressed throughout the brain, including several areas controlling food intake and neuroendocrine function such as the hypothalamus and the hindbrain, with immunostaining revealing a mainly neuronal distribution of the various AMPK isoforms (10). Hypothalamic AMPK is influenced by energy intake and availability, as demonstrated by the fact that fasting increases and re-feeding decreases its activity in the hypothalamus (11). It is also affected by several orexigenic and anorexigenic signals in the hypothalamus (Table 1) (7).

**Table 1:** Compounds and Signals that Affect Hypothalamic AMP-Activated Protein Kinase (AMPK) Activity.

Orexigenic signals (activators of hypothalamic AMPK)	Anorexigenic signals (inhibitors of hypothalamic AMPK)
Hypoglycaemia, 2-deoxyglucose (14–17)	Glucose (11, 14)
Agouti-related peptide (11)	Leptin (11)
Ghrelin (12, 27)	Insulin (11)
Cannabinoids (27)	C75 (46)
Glucocorticoids	α-Lipoic acid (14)
Adiponectin (42)	Metformin (17)
Low temperatures (53)	Ciliary neurotrophic factor analogue (20)
Thyroid hormones (50)	α-melanocyte-stimulating hormone, MT-II (a melanocortin 4 receptor agonist) (11, 73)

## Effects of pharmacological/genetic modifications of hypothalamic AMPK on food intake

The first study to report the involvement of AMPK in appetite regulation showed that activation of AMPK via injection of 5-amino-4-imidazole carboxamide riboside (AICAR), a pharmacological activator of AMPK, into either the third ventricle or directly into the paraventricular nucleus of the hypothalamus significantly increased food intake (12). In a very meticulous study, Minokoshi *et al.* (11) showed that the expression of dominant negative AMPK in the hypothalamus was sufficient to reduce food intake and body weight, whereas constitutively active AMPK increased both. Although these first two studies and numerous following studies confirmed the involvement of the hypothalamic AMPK in the mediation of the appetite-stimulating or appetite-inhibiting effects of various agents, a recent study by Claret *et al.* (13) questioned some of these concepts and suggested that more detailed cell- or hypothalamic nucleus-specific studies are needed. Most of the studies performed to date have studied AMPK activity either in whole hypothalamus or in specific nuclei. In addition, many studies have used AICAR as an activator of AMPK and Compound C as an inhibitor. Several studies now suggest that neither AICAR, nor Compound C are specific to AMPK and other relevant related pathways could be influenced by these pharmacological agents (4); therefore, studies using dominant negative or constitutively active AMPK mutants or the most recent studies with cell-type specific silencing of AMPK could provide a novel insight. To explore in more detail the specific hypothalamic neurones where AMPK plays a role, α2 AMPK was specifically knocked out in hypothalamic Agouti-related peptide (AgRP) neurones or in hypothalamic pro-opiomelanocortin (POMC) neurones via crossing mice with floxed α2 AMPK gene with mice harbouring the Cre recombinase in AgRP and POMC promoters (13). AgRP α2 AMPK-KO showed decreased body weight even though there were no changes in food intake or energy expenditure and, the difference in body weight was lost when the animals were fed a high fat diet (HFD). POMC α2 AMPK-KO animals, showed unexpectedly increased body weight and adiposity, which was further enhanced by a HFD. These findings could not be explained by baseline morphological changes in the neurones or by the possible studied compensatory mechanisms. AMPK is known to undergo tissue-specific regulation and these findings point to a cell-specific role for AMPK. Losing AMPK in orexigenic (AgRP) neurones leads to reduced body weight whereas loss of the enzyme in anorexigenic (POMC neurones) leads to increased body weight.

## Energy-related AMPK changes

AMPK activity is regulated by intracellular energy levels, but it also senses whole body energy levels because it is affected by short-term (fasting and feeding acutely) and long-term energy signals (chronic changes in diet-induced obesity (DIO). Fasting results in activation of AMPK whereas re-feeding inhibits AMPK activity in multiple hypothalamic regions in mice (11). Fasting and re-feeding are characterised by changes in nutrients (e.g. glucose) and hormones (e.g. insulin) levels, which could explain the effect on hypothalamic AMPK. Several studies have demonstrated that hypothalamic AMPK is regulated by blood glucose levels. Peripheral or central hyperglycaemia inhibits AMPK in the arcuate nucleus (ARC), the ventro- and dorso-mediobasal hypothalamus (VMH and DMH), the paraventricular nucleus (PVN) and the lateral hypothalamus (LH) (11, 14). Insulin-induced hypoglycaemia and inhibition of intracellular glucose utilisation through the administration of 2-deoxyglucose (2-DG) both increase hypothalamic AMPK activity and food intake (14, 15). Concordantly, in neuronal cell lines and *ex-vivo* hypothalamic cultures, low glucose and 2-DG increase hypothalamic AMPK activity, similarly to the AMPK activator AICAR (16, 17), whereas high glucose and pyruvate supplementation in 2-DG treated-cells decrease expression and phosphorylation of AMPK through changes in intracellular ATP levels (16). Long-term energy signals also appear to affect hypothalamic AMPK. DIO mice have suppressed AMPK activity in the PVN of the hypothalamus (18). The lower basal AMPK activity in PVN may be due to effects of hyperinsulinaemia and/or hyperglycaemia, which suppress AMPK activity in multiple hypothalamic nuclei (11, 15). AMPK is suppressed to the level observed in leptin-treated chow-fed mice, and there is no further effect of leptin (18). In mice, DIO alters the effect of leptin on AMPK activity not only in the hypothalamus, but also in the skeletal muscle (18, 19). However, when the ciliary neurotrophic factor analogue (CNTF_Ax15_), whose signalling pathway in the hypothalamus partially overlaps with that of leptin, is given i.c.v. it not only reduces food intake of DIO mice, but also further suppresses hypothalamic AMPK activity, bypassing diet-induced leptin resistance (20). Diabetic rats have enhanced AMPK activity, despite their high glucose levels, which should suppress hypothalamic AMPK. The activation of AMPK could contribute to their hyperphagia and may be explained by the lower plasma levels of leptin and insulin in these animals (21).

## AMPK as a mediator of several orexigenic and anorexigenic signals

Leptin has a tissue-specific effect on AMPK: in the skeletal muscle, it stimulates AMPK activity whereas, in the hypothalamus, it has the opposite effect, decreasing hypothalamic AMPK activity (11, 12, 22). These apparently paradoxical tissue-specific effects of leptin contribute both to the overall positive effect of leptin on energy homeostasis, leading to increased fatty acid oxidation in peripheral tissue (23, 24) and to reduced appetite in the hypothalamus with consequent reduction of body weight. Minokoshi *et al.* (11) studied the effect of leptin in the specific hypothalamic nuclei and showed that leptin inhibits AMPK activity in ARC and PVN. Furthermore, inhibition of hypothalamic AMPK is necessary for leptin’s effects on food intake and body weight, as constitutively active AMPK blocks these effects. A more recent study confirmed the importance of AMPK in the effect of leptin as AICAR was able to reverse the inhibitory effect of leptin on the electrical activity of glucose-inhibited neurones (25). However, the selective AMPKα2-deficient POMC or AgRP neurones showed normal response to leptin in another study (13).

I.c.v. MT-II, a melanocortin 4 receptor agonist, was shown to decrease AMPK activity in PVN, whereas AgRP, a melanocortin receptor antagonist, increased AMPK activity. Insulin i.c.v. inhibits AMPK activity in multiple hypothalamic regions 3 h after injection (11).

Ghrelin is a circulating growth hormone-releasing and appetite-inducing brain-gut peptide with predominant expression in the gastric mucosa, but low-level widespread expression throughout the body (26). Ghrelin stimulates hypothalamic AMPK following i.p. injection (12, 27) or i.c.v. injection (27), suggesting that AMPK activation might be part of its orexigenic effect. In addition, ghrelin has direct peripheral actions in several organs, including the liver and adipose tissue, and similar to leptin, has an opposite (inhibitory) effect on the AMPK in these tissues (27).

To date, there is no explanation as to why some hormones have tissue-specific effects on AMPK activity. This could possibly be due to different AMPK upstream kinases or AMPK phosphatases being affected in different tissues.

The upstream kinase by which ghrelin exerts its effects on AMPK activity has not yet been reported. The only receptor so far identified for ghrelin is the growth hormone secretagogue receptor type 1a, which is a Gq-PKC pathway-coupled receptor (28), and ghrelin has been shown to induce Ca^2+^ signalling in neuropeptide Y (NPY) neurones in the ARC (29, 30). CaMKKβ is regulated by intracellular Ca^2+^ levels (3, 31) and therefore constitutes a possible candidate in mediating the effect of ghrelin on AMPK activity.

Both exogenous and endogenous cannabinoids stimulate appetite in the hypothalamus via cannabinoid receptor type 1 (CB1) (32–34). Furthermore, endocannabinoid hypothalamic levels change in response to fasting and feeding (35) and seem to play an important role in mediating the anorexigenic effects of leptin (36) and ghrelin (37). We have shown that cannabinoids stimulate AMPK activity in the hypothalamus, and this could explain their central orexigenic effects (27). By contrast, they inhibit AMPK activity in the liver and adipose tissue, which may lead to fuel, particularly fat, storage (27). The combined effect of both central and peripheral signals would therefore be increased food intake and lipid storage, leading to lipid deposition and weight gain.

**Fig. 1:**
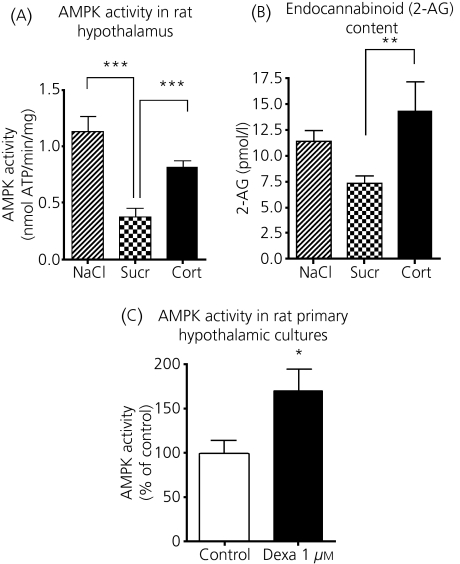
Effects of glucocorticoid treatment on hypothalamus. Hypothalamic AMP-activated protein kinase (AMPK) (a) and hypothalamic endocannabinoid (2-AG) content (b) in a rat model of Cushing’s syndrome (corticosterone pellet for 2 weeks with sucrose-drinking, n = 6 per group) and appropriate control groups (placebo pellet with saline- or sucrose-drinking). (c) AMPK activity in primary hypothalamic cultures treated with dexamethasone 1 μm for 6 h compared to control treatment (expressed as % of control). *P < 0.05; **P < 0.01; ***P < 0.001 Modified from Christ-Crain *et al.* (38).

Glucocorticoids are also known to increase appetite and we have shown in a rodent model of Cushing’s syndrome that the features of the metabolic syndrome are associated with increased AMPK activity in the hypothalamus (Fig. 1) (38). Corticosterone-treated animals had *ad libitum* sucrose available to block the marked catabolism caused by high-dose corticosterone-pellets. Sucrose-drinking animals had lower hypothalamic AMPK activity compared to saline-drinking control rats, confirming the inhibitory effect of sucrose on AMPK activity. Corticosterone administration counteracted this effect of sucrose and increased hypothalamic AMPK activity to levels comparable with saline-drinking animals (Fig. 1a). The effect of glucocorticoids on hypothalamic AMPK is possibly mediated by endogenous cannabinoids because 2-arachydol-glycerol (2-AG) (Fig. 1b) and anandamide contents were higher in corticosterone-treated rats than in sucrose drinking control rats. We also showed a dexamethasone-induced increase in AMPK in primary rat hypothalamic cell cultures, suggesting a direct effect of glucocorticoids on AMPK activity (Fig. 1c). Metformin, which has previously been shown to inhibit hypoglycaemia-induced AMPK (17), was also able to inhibit the stimulatory effect of dexamethasone in primary hypothalamic culture (38).

Adiponectin, an adipokine secreted exclusively by the white adipose tissue, has been shown to stimulate fatty acid oxidation through the activation of AMPK in the peripheral tissues (39–41). However, a recent study shows that peripheral adiponectin also has central effects in the hypothalamus as its trimer and hexamer forms can enter the cerebrospinal fluid (42). Surprisingly, and apparently in disagreement with its well-assigned role as an anti-diabetic hormone, adiponectin enhances AMPK activity in the ARC after re-feeding, stimulates food intake and decreases energy expenditure (42). Conversely, adiponectin-deficient mice showed decreased AMPK phosphorylation in the ARC, decreased food intake, and increased energy expenditure, exhibiting resistance to high fat diet-induced obesity. Serum and cerebrospinal fluid adiponectin levels increased during fasting and decreased during re-feeding, suggesting a role for adiponectin as a starvation hormone. In this guise, under fasting conditions, high adiponectin levels would stimulate AMPK and food intake and would decrease energy expenditure, promoting fat storage. After re-feeding adiponectin levels would fall with a consequent decrease in AMPK activity and food intake and an increase in energy expenditure. Under fasting or after re-feeding conditions, adiponectin levels correlate inversely to serum leptin levels, and because leptin’s effect on hypothalamic AMPK and food intake is apparently opposite to that of adiponectin, Kubota *et al.* (42) conclude that a joint signal of the two hormones regulates whole body energy homeostasis under these conditions. These important findings contrast with previous data on the role of peripheral adiponectin and point to a dissociated role between peripheral adiponectin and central nervous system adiponectin. Hormones such as leptin and ghrelin have opposite, tissue specific effects in the hypothalamus and the peripheral organs (7), whereas adiponectin appears to have a stimulatory effect on the enzyme in all the different tissues studied. This very exciting study points out once more the complexity of the systems that regulate whole body metabolism and the necessity for further research in this direction.

Metformin, an important anti-diabetic agent, stimulates AMPK in the liver and in the muscle (43–45). In analogy with other hormones, metformin has an opposite effect on AMPK in the hypothalamus. Chau-Van *et al.* (17) showed that metformin blocks the AMPK phosphorylation induced by low glucose in primary cultures of rat hypothalamic neurones. Consistently, metformin inhibited NPY mRNA expression induced by low glucose conditions, whereas no effect was present on POMC gene expression. These data could provide a potential mechanism of action for the anorectic effects of metformin (17).

C75, a fatty acid synthase (FAS) inhibitor, which causes weight loss and anorexia, rapidly reduced the level of the phosphorylated AMPKα subunit in the hypothalamus (46). It also reduced pAMPK levels in fasted mice that had elevated hypothalamic pAMPK. AICAR was able to reverse both the inhibitory effect on pAMPK and the C75-induced anorexia (46).

α-Lipoic acid, an antioxidant that reduces food intake, also inhibits AMPK activity in the hypothalamus (14). AICAR or expression of a constitutively active AMPK variant prevent its anorexigenic effect (14).

Although there is evidence that thyroid hormones stimulate AMPK and acetyl-CoA carboxylase (ACC) expression in rat skeletal muscle (47, 48) and propylthiouracil (an inhibitor of thyroid hormone synthesis) inhibits this effect (49), there are no conclusive data about their effect on hypothalamic AMPK. Preliminary data have shown that triiodothyronine increases AMPK activity in the hypothalamus, offering a possible explanation for the hyperphagia observed in hyperthyroid states (50).

The role of AMPK as an appetite mediator has also been investigated in conditions known to affect food intake such as exercise and low temperatures.

Exercise is known to influence appetite, but changes in energy metabolism during or after exercise are probably not coordinated by changes in hypothalamic AMPK as 1 h of strenuous exercise in rats did not elicit significant changes in hypothalamic AMPK activity despite an increase in plasma ghrelin (51). Other exercise-induced cytokines (e.g. IL-6) may also have had an effect on hypothalamic AMPK and might have opposed the effect of increased plasma ghrelin (52). The effects of exercise on food intake are also exercise-type and time-dependent. Unfortunately, the effects of exercise on food intake were not reported in this study (51).

Interestingly, AMPK has been shown to mediate cold-induced resistance to anorexigenic signalling in the hypothalamus (53). Feeding and insulin-induced anorexigenic responses are reduced by exposure to cold, probably due to the cold-induced AMPK activation and ACC inactivation. The insulin-induced inhibition of AMPK is also impaired by cold-exposure. This study offers a plausible explanation for the increased feeding noticed during cold-exposure which may result, at least in part, from resistance to insulin and nutrient-dependent anorexigenic signalling in the hypothalamus (53).

## Downstream mediators of AMPK in hypothalamus

**Fig. 2:**
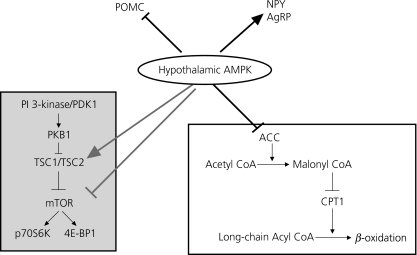
Downstream targets of AMP-activated protein kinase (AMPK) in the hypothalamus. The effect of AMPK on the mammalian target of rapamycin (mTOR) pathway (grey box and grey arrows) has not been directly proven in the hypothalamus. AMPK, AMP-activated protein kinase; POMC, pro-opiomelanocortin; NPY, neuropeptide Y; AgRP, Agouti-related peptide; ACC, acetyl-CoA carboxylase; CPT1, carnitine palmitoyltransferase 1 (CPT1).

The mechanisms through which AMPK influences feeding behaviour are not fully understood (Fig. 2). Changes in the expression of well-known orexigenic and anorexigenic genes, such as NPY, POMC and AgRP, have been reported with modulation of AMPK activity. Alterations of hypothalamic AMPK activity augment changes in arcuate neuropeptide expression induced by fasting and feeding (11). Overexpressing DN-AMPK in mediobasal hypothalamus suppresses mRNA expression of orexigenic neuropeptides, NPY and AgRP in ARC, whereas overexpressing CA-AMPK enhances the fasting-induced increase in expression of NPY and AgRP in ARC and melanin-concentrating hormone in the lateral hypothalamus (11).

Low glucose levels in neuronal cell lines not only increased phosphorylation of AMPK, but also increased AgRP expression (16). High glucose concentrations in *ex vivo* hypothalamus culture decreased expression of both AgRP and NPY, whereas pyruvate supplementation suppressed 2-DG-induced AgRP expression (16). Glucose has also been shown to inhibit AMPK activity in a POMC-expressing hypothalamic cell line, with a resulting increase in expression of POMC mRNA (54). By contrast, metformin, which blocks the AMPK phosphorylation induced by low glucose, does not affect POMC gene expression, but does inhibit the stimulation of NPY observed in low glucose conditions (17). These changes in neuropeptide expression are very likely to mediate, at least in part, the effects of AMPK in appetite regulation.

The downstream pathways of AMPK in the hypothalamus may involve the ACC-malonyl-CoA-carnitine palmitoyltransferase 1 (CPT1) pathway and the mammalian target of rapamycin (mTOR) pathway. Some studies show that modulation of AMPK by leptin (12), α-lipoic acid (14) or ghrelin (27) is associated with decreased (leptin and α-lipoic acid) or increased (ghrelin) phosphorylation of its downstream target ACC, leading to stimulation or inhibition of ACC activity (Fig. 2). Activation of ACC, as a consequence of AMPK inhibition, would lead to increased intracellular malonyl-CoA levels, which would inhibit mitochondrial CPT1 and fatty acid oxidation. In support of this mechanism, degradation of malonyl-CoA in the mediobasal hypothalamus of rats results in increased food intake and progressive weight gain (55), whereas inhibition of CPT1 in the hypothalamus suppresses food intake (56, 57).

The anorexigenic effect of leptin is mediated by inhibition of AMPK, probably in a coordinate manner with the signal transducer and activator of transcription (STAT3) and PI3K pathways (11). The activation of the hypothalamic STAT3 pathway is an absolute requirement for the effects of leptin on food intake (58, 59). Leptin increases STAT3 phosphorylation in all hypothalamic regions, whereas its effect on AMPK activity is limited to ARC and PVH (11). Furthermore, the effects of DN- and CA-AMPK could not be explained by alterations in STAT3 tyrosine phosphorylation or protein level in the hypothalamus, suggesting that AMPK functions either downstream of, or in a parallel pathway to, STAT3 to modulate its effects on food intake (11). Similarly to leptin, the effect of CNTFAx15 on food intake and exspression of orexigenic peptides is mediated by increased STAT3 phosphorylation in the ARC and suppressed AMPK activity (20). Interestingly, SOCS3, an inhibitor of leptin-STAT3 signalling, inhibits leptin activation of AMPK in primary myotubes (60).

Another possible downstream target of AMPK in the hypothalamus is the mTOR pathway. AMPK affects protein synthesis in the periphery through the mTOR-P70S6-kinase pathway (61). In the hypothalamus, a direct link between the AMPK and mTOR has not been proven but is tempting to think so because central treatments with both leptin and leucine have been found to inhibit food intake and reduce body weight through stimulation of this pathway (62).

## Role of AMPK in hypothalamic glucose sensing

**Fig. 3:**
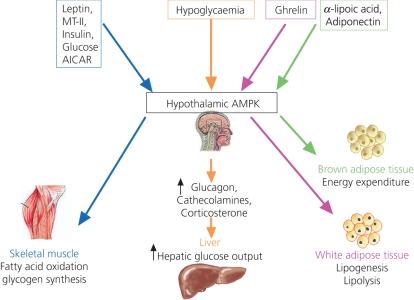
Effects of different central treatments on peripheral metabolism through changes in hypothalamic AMP-activated protein kinase (AMPK). Intracerebroventricular leptin, MT-II, insulin, glucose and 5-amino-4-imidazole carboxamide riboside (AICAR) treatment affect skeletal muscle metabolism through hypothalamic AMPK (blue arrows). Hypoglycaemia stimulates hypothalamic AMPK and exerts a consequent counter-regulatory response with a final effect on hepatic glucose output (orange arrows). Central ghrelin influences white adipose tissue AMPK activity (purple arrows) and central adiponectin and α-lipoic acid administration change energy expenditure in brown adipose tissue (green arrows).

Several studies have investigated the role of AMPK in glucose sensing by specialised glucose-sensing neurones in the hypothalamus and in the hormonal counter-regulatory response to hypoglycaemia. Appropriate counter-regulatory response is crucial for recovery from hypoglycaemia and AMPK activation appears to mediate this function (Fig. 3). Insulin-induced hypoglycaemia in rats increases AMPK phosphorylation and α2AMPK activity in the ARC/VMH and PVN (15). Inhibition of hypothalamic AMPK, either via Compound C or dominant negative AMPK expression, inhibits the hypoglycaemia-induced increase in the counter-regulatory hormones glucagon, corticosterone and catecholamines, causing a severe and prolonged hypoglycaemia (15). Consistent with this finding, VMH AMPK down-regulation, via virus-assisted expression of short hairpin RNA for αAMPK, resulted in suppressed glucagon and epinephrine responses to acute hypoglycaemia (63). These rats required more exogenous glucose to maintain glucose levels and showed significant reductions in endogenous glucose production (63). AICAR administration in the VMH during a hyperinsulinaemic-hypoglycaemic clamp resulted in a significant reduction in the amount of exogenous glucose required by the rat to maintain the hypoglycaemic plateau (64). This effect is associated with a three- to four-fold increase in hepatic glucose output, suggesting that the combination of hypoglycaemia- and AICAR-induced AMPK activity results in a marked stimulus to hepatic glucose production (64).

Recurrent hypoglycaemia can lead to a phenomenon known as hypoglycaemia-associated autonomic failure (65). In this situation, antecedent hypoglycaemia blunts the counter-regulatory hormone response that normally restores normo-glycaemia (66). Single i.c.v. injections of 2-DG increased both α1- and α2-AMPK activities in ARC at 10 min, and increased AMPK activity persisted at 60 min (67). When 2-DG was injected i.c.v. once a day for 4 days, inducing recurrent neuroglucopaenia, hypothalamic α1- and α2AMPK responses were markedly blunted at 10 min whereas normal activation was seen at 60 min, suggesting impaired or delayed AMPK activation. Impaired counter-regulatory hormone responses were partially restored by i.c.v. injection of AICAR before the 2-DG (67). In another study, 3 days of recurrent insulin-induced hypoglycaemia resulted in an increase in the gene expression of α1- and α2AMPK in the whole hypothalamus and in the VMH (68). Phosphorylation status of AMPK was not reported in this study and the different model of recurrent hypoglycaemia used could explain the apparent differences in results between the two studies (67, 68). VMH injection of AICAR was similarly found to amplify the counter-regulatory response (68).

## Role of AMPK in the determination of hypothalamic neuronal electrical activity

In addition to the effect of AMPK on the counter-regulatory response control, AMPK also mediates the effects of glucose, and possibly other hormones, on the electrical activity of hypothalamic neurones.

Two types of glucose-sensing neurones have been identified. Activity of glucose-inhibited (GI) neurones decreases in response to increased glucose concentrations whereas glucose-excited neurones increase their activity in response to increased glucose concentrations (69). GI neurones are present in the VMN, in the ARC and in the LH and partly overlap with the orexigenic NPY/AgRP and possibly orexin neurones. GE neurones correspond in part to POMC neurones in the VMN and possibly in ARC and to MCH neurones in the LH (69). Mountjoy *et al.* (25) showed that, in GI neurones of the basomedial hypothalamus (40% of NPY-expressing neurones are GI neurones), the effect of large fluctuations in glucose concentration on electrical activity and Ca^2+^ oscillation frequency is mediated through changes in AMPK activity. The stimulatory effects of decreased glucose on GI neurones were mimicked by AICAR and blocked by Compound C. Similar data were shown with leptin. Leptin suppressed the activity of GI neurones induced by low glucose concentrations and this inhibitory effect was reversed by AICAR in the majority of GI neurones (25). Forced changes in AMPK activity, by application of either AICAR or Compound C, had no effect on glucose-excited and non-glucose-responsive neurones (25). Based on these studies, it has been suggested that AMPK plays a role in the glucose sensing effect of GI neurones but not in GE neurones. Claret *et al.* (13) showed that ARC POMC neurones are hyperpolarised (inhibited) by reductions in extracellular glucose concentration. Surprisingly, and in contrast with previous studies (70, 71), a minority of ARC AgRP neurones were also inhibited by decreasing glucose levels but not affected by increasing glucose concentrations (13). POMC α2 AMPK-KO and AgRP α2 AMPK-KO neurones did not change their firing rates or their membrane potential in response to glucose changes, suggesting a role for AMPK as a common glucose-sensor in these neurones (13). By contrast, the depolarising effect of leptin and the repolarising effect of insulin in a minority of ARC POMC were maintained in POMC α2 AMPK-KO neurones. Leptin did not have an effect on AgRP neurones whereas insulin depolarised AgRP neurones and the effect was still present in AgRP α2 AMPK-KO neurones. These data suggest AMPK-independent pathways for these two anorexigenic hormones. This is partly in contrast with other studies that have reported AMPK dependent effects of leptin (25). It is important to emphasise that GE neurones do not overlap completely with POMC neurones in the ARC while the GI neurones do not completely overlap with NPY/AgRP neurones. Indeed, Claret *et al.* (13) found that alterations in external glucose levels did not alter the excitability of all ARC POMC and AgRP neurones. The different results may be explained, in addition to the different techniques and experimental setups, by the presence of distinct subpopulations of GE and GI neurones with different neuropeptide phenotypes, responses to hormonal stimuli and, in some cases, different glucose-sensing mechanisms (69). Future studies are needed to clarify some of these contrasting data.

AMPK activity also influences NO production and neuronal activity in GI neurones. The AMPK activator, AICAR, increases both NO production and neuronal activity in GI neurones of the VMH (72). Glucose and leptin suppress neuronal nitric oxide synthase-dependent NO production in cultured VMH GI neurones, whereas insulin stimulates it (72). The effects of decreased glucose and leptin are blocked by inhibition of AMPK with Compound C, whereas the effect of insulin is AMPK-independent. These data further suggest that the effects of glucose and leptin in GI neurones are mediated by the suppression of AMPK.

## Role of hypothalamic AMPK in regulating peripheral metabolism

One of the most interesting findings of the recent research into hypothalamic regulation of appetite is the neuronal link between the hypothalamus and the periphery. The hypothalamic regulation of peripheral metabolism that had previously been recognised seems now to extend to the AMPK as well (Fig. 3). Minokoshi *et al.* (22) described that leptin treatment affects skeletal muscle AMPK both directly and via the hypothalamic-sympathetetic nervous system axis. The endogenous melanocortin system is implicated in this effect because the central melanocortin receptor agonist MT-II increases and melanocortin receptor antagonist SHU9119 decreases AMPK phosphorylation in the skeletal muscle. This effect is independent of their effect on food intake, as shown in pair-fed animals (73).

In leptin-over-expressing transgenic mice on a HFD, muscle AMPK phosphorylation and ACC phosphorylation are reduced compared with standard diet leptin-over-expressing transgenic mice and are comparable to HFD-non-transgenic mice. In these animals, leptin i.c.v., in addition to transgenic hyperleptinaemia, is not able to restore the impaired AMPK signalling because of the induced generalised leptin resistance. However, MT-II i.c.v. significantly augments AMPK and ACC phosphorylation, suggesting that MT-II is a potent AMPK activator in muscle, even in mice on a HFD (73). Central adiponectin treatment also has effects in the periphery: i.c.v. injection of adiponectin results in decreased energy expenditure, possibly as a result of a reduced expression of uncoupling protein-1 (UCP-1) in brown adipose tissue (42). Central α-lipoic acid, which inhibits hypothalamic AMPK activity, increases energy expenditure and UCP-1 expression in brown adipose tissue (14). Co-administration of i.c.v. AICAR prevents the effects of α-lipoic acid (14). We have obtained preliminary data suggesting that i.c.v. treatment with ghrelin affects adipose tissue AMPK activity (B. Kola, M. Christ-Crain, G. Wittmann, F. Lolli, F. Amin, A. Grossman, C. Fekete, M. Korbonits, unpublished data). This effect is supported by another study showing that chronic central ghrelin treatment results in increased glucose utilisation rate of white and brown adipose tissue; these effects are independent of ghrelin-induced hyperphagia (74). Central ghrelin treatment has also been shown to inhibit, at least in part, independently from the effect on food intake, the effects of i.c.v. leptin treatment on fat weight, plasma insulin and glucose (75).

Interestingly, hypothalamic AMPK has also been implicated in the regulation of muscle glycogen synthesis as i.c.v. AICAR treatment increased both insulin-mediated and non insulin-mediated glycogen synthesis (76). Central insulin infusion also increased muscle glycogen synthesis. This effect was blocked by the co-administration of glucose (76).

Hypothalamic AMPK, as described above, also mediates the counter-regulatory response to hypoglycaemia, increasing the release of peripheral hormones such as corticosterone, catecholamines and glucagon (15, 67, 68).

These data emphasise the complexity of the regulation of whole body metabolism, and the role of AMPK being not only a peripheral or a central mediator, but also a key enzyme in coordinating the interaction between peripheral and central energy regulation.

## Conclusions

Despite great advances in our understanding of hypothalamic control of food intake in recent years, many questions remain unanswered. It appears that AMPK plays an important role in appetite regulation, with increased AMPK activation resulting in increased food intake, decreased energy expenditure and, predictably, increased body weight. The converse is also seen with reduced AMPK activity causing decreased food intake. However, these experimental models differ and any direct comparisons must be made with caution. The effect of each of the treatments discussed on AMPK varies according to the tissue type, the duration of exposure and the feeding conditions of the animal. For example, leptin and metformin stimulate AMPK activity in skeletal muscle but inhibit it in the hypothalamus, whereas ghrelin and cannabinoids stimulate AMPK in the hypothalamus but inhibit it in liver and adipose tissue (7). Even within the hypothalamus itself, there is localisation of the effect of different compounds on AMPK: leptin acting in the ARC and PVN, glucose and insulin in the mediobasal and lateral hypothalamus as well as the ARC and PVN (11). AMPK appears to have an alternative role in the hypothalamic neurones because AMPK loss in AgRP neurones leads to reduced body weight, whereas its loss in POMC neurones leads to increased appetite and body weight (13), suggesting a cell-specific role for this enzyme. The complexities of this system are further demonstrated by some hormones affecting hypothalamic AMPK in *ad libitum* feeding conditions [e.g. ghrelin or cannabinoids (27)], whereas others only have an effect after variable time of fasting or re-feeding [e.g. leptin (11), adiponectin (42)]. A further complication could arise from the fact that the effect of AMPK activators and inhibitors (AICAR, metformin and Compound C) may not be specific to AMPK (4). Therefore, the results obtained from studies using these agents should be interpreted with caution. Appetite regulation and whole body energy homeostasis remain challenging physiological processes and much research is still needed in this field. In particular, very little is known about the intermediate players acting between hormones, their receptors and AMPK and its upstream regulation. More work is also needed to unravel the exact mechanisms by which AMPK status affects food intake.
